# Optical Sensing Using Fiber-Optic Multimode Interference Devices: A Review of Nonconventional Sensing Schemes

**DOI:** 10.3390/s21051862

**Published:** 2021-03-07

**Authors:** José Rafael Guzmán-Sepúlveda, Rafael Guzmán-Cabrera, Arturo Alberto Castillo-Guzmán

**Affiliations:** 1 Centro de Investigación y de Estudios Avanzados del IPN, Unidad Monterrey, Vía del Conocimiento 201, Parque de Investigación e Innovación Tecnológica, km 9.5 de la Autopista Nueva al Aeropuerto, Apodaca, 66600 Nuevo León, Mexico; 2 Departamento de Ingeniería Eléctrica, División de Ingenierías, Campus Irapuato-Salamanca, Universidad de Guanajuato, km 3.5 + 1.8 carretera Salamanca-Valle de Santiago, Salamanca, 36730 Guanajuato, Mexico; guzmanc@ugto.mx; 3 Facultad de Ciencias Físico Matemáticas, Universidad Autónoma de Nuevo León, San Nicolás de los Garza, 66455 Nuevo León, Mexico; arturo.castillogz@uanl.edu.mx

**Keywords:** fiber-optic sensors, multimode interference phenomena, optical sensing

## Abstract

We review fiber-based multimode interference (MMI) devices with a particular focus on optical fiber-based sensing applications. The present review complements a recently published, extensive review where the sensing of conventional physical variables such as refractive index, temperature, displacement, and strain was covered. This review focuses on MMI fiber sensors for nonconventional physical variables, including mechanical, electromagnetic, chemical, and optical, covering around fifteen years of work in the field. Finally, by the end of this paper, we also review some new trends of MMI-based schemes based on polymer fibers, for wavelength-locking applications, for retrieving the thermo-optic coefficient of liquid samples, and for measuring the dynamics of complex fluids.

## 1. Introduction

The self-imaging phenomenon was first observed by Talbot [1] in the context of the diffraction from gratings and, later on, explained by Rayleigh [2]. Since then, the field has consolidated until a solid understanding of the subject has been achieved [3,4,5,6]. 

For some time, self-imaging phenomena were restricted to image-formation free-space applications [7,8]. However, it was soon recognized that, as self-imaging results directly from Fresnel diffraction, these effects could be observed in waveguides supporting multiple modes. This fact opened new technological paths that allowed for the development of photonic integrated devices based on these multimode interference (MMI) effects [9,10]. Examples of these are N-by-N couplers/splitters on different material platforms, asymmetric splitters for power monitoring, polarization beam splitters, and devices based on biopolymer waveguides. 

Regarding optical fiber technology, self-interference phenomena were initially used to optimize the transmission through fiber links. Later on, optical fiber-based devices with more flexible functionalities, such as tunable fiber lenses [11], band-pass filters [12], and a wide variety of sensors and tunable lasers [13], started being developed.

**Figure 1:**
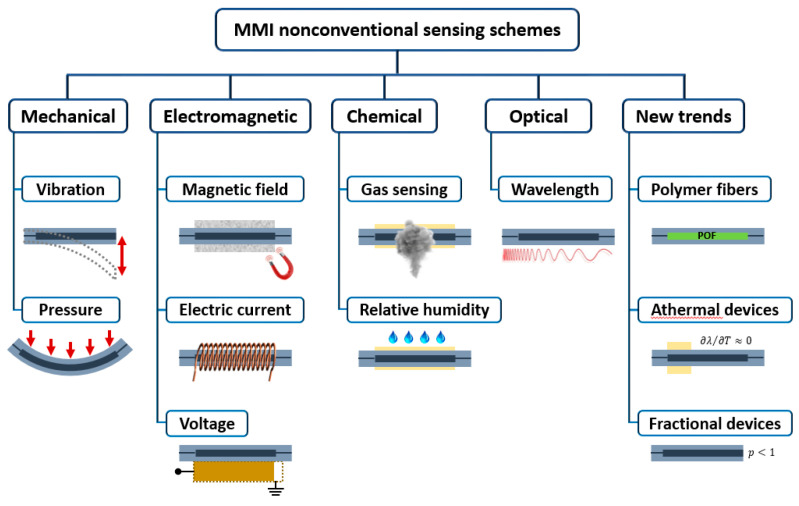
Schematic overview of the structure of the present review.

In this work, we review optical-fiber-based MMI devices with a particular focus on sensing applications. The present review complements another extensive review, recently published by the group of Prof. Koch [14]. In their paper, Prof. Koch and collaborators reviewed MMI fiber devices for sensing applications of ‘conventional’ physical variables such as refractive index (RI), temperature, displacement, and strain. As we will see later, these physical variables directly determine the spectral response of an MMI device; some of them even appear explicitly in the equations. However, several other physical variables can be sensed indirectly using MMI fiber devices whose manifestations are implicit. This review focuses on MMI fiber sensors for such ‘nonconventional’ physical variables, including mechanical variables such as vibration and pressure; electromagnetic variables such as voltage, electric current, and magnetic field; chemical variables such as relative humidity and gas concentration; and optical variables such as the wavelength. By the end of this review, we also approach some new trends and applications of MMI-based schemes based on polymer fibers, for wavelength-locking applications, for retrieving the thermo-optic coefficient (TOC) of liquid samples, and for measuring the dynamics of complex fluids. Figure 1 illustrates the structure and overview of the present review.

## 2. Fundamentals and Background of Fiber-Based MMI Devices

**Figure 2:**
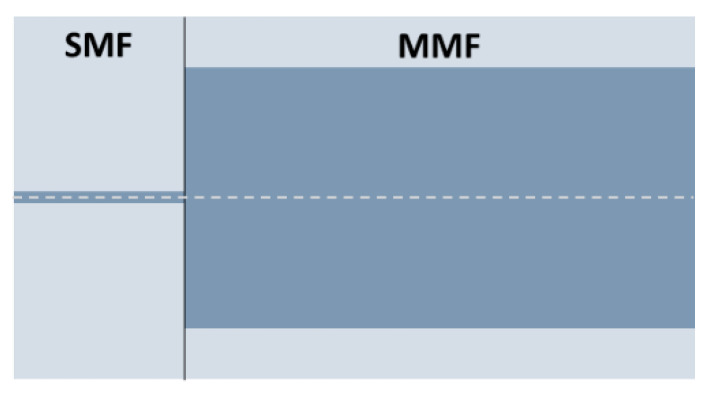
Single-mode fiber (SMF)-multimode fiber (MMF) structure used in the simulations to illustrate the main features of multimode interference (MMI) devices.

In order to show the main features of MMI devices, we simulated a standard structure consisting of a single-mode fiber (SMF) spliced to a section of multimode fiber (MMF), as shown schematically in Figure 2, by following the formalism given in Refs. [11,15]. The two fibers are assumed to be coaxial without any lateral offset between their centers. The strategy consists of calculating the energy coupled from the excitation field (SMF) to each of the modes supported by the MMF; then, all the modes, each with its own propagation constant, are propagated along the MMF; and finally, at each propagation distance, the total field is calculated as the coherent superposition, i.e., on a field basis, of all the modes. All these steps are analyzed in detail in the following sections.

### 2.1. Interference between Multiple Modes—Energy Coupling

In the SMF-MMF arrangement considered, both optical fibers have a step-index profile, and the fibers are aligned about the propagation axis, i.e., there is no lateral offset between the cores of the SMF and the MMF. As mentioned before, the input field determines the set of modes supported by the MMF that are actually excited based on the energy transfer to each of these modes. In general, the superposition of all the modes excited at z=0 (at the input of the multimode waveguide) can be written as:

(1) $$E\left(x,y,0\right)=\underset{m}{\sum }c_{m}F_{m}\left(x,y\right)$$

where $F_{m}\left(x,y\right)$ and $c_{m}$ are the spatial field distribution and the field excitation coefficient of the m-th eigenmode, respectively.

At this point, we would like to clarify some subtle issues that are often overlooked in the literature. For instance, the terms MMI and self-imaging are sometimes used interchangeably; however, self-imaging is a particular case of the MMI phenomena that takes place under restricted conditions of high symmetry, and refers to the periodic repetition of the input field in propagation as a result of the interference of the multiple modes. In general, one always has MMI if multiple modes are excited in the MMF, but that does not imply that the conditions for self-imaging are satisfied. Typically, MMI devices are operated under self-imaging conditions because that gives rise to a filter-like spectral response when a continuum spectrum is used for the excitation. This will be addressed in more detail below. 

For the case of a circularly symmetric waveguide such as an optical fiber, where the excitation field imposes a radial symmetry, i.e., by the fundamental mode supported by the SMF, all the modes excited also satisfy this restriction of radial symmetry by virtue of the overlap integral from where the energy transfer is calculated. Thus, for a radially symmetric situation, the superposition of excited modes from Equation (1) can be expressed in terms of only the radial coordinate as:

(2) $$E\left(r,0\right)=\underset{m}{\sum }c_{m}F_{m}\left(r\right)$$

The set of eigenfunctions $F_{m}\left(r\right)$ correspond to radially symmetric field distributions (${\mathrm{LP}}_{0m}$ modes), which can be expressed in terms of Bessel functions as:

(3) $$F_{m}\left(r\right)=\{\begin{array}{c} c_{m}^{\left(1\right)}\;J_{0}\left(u_{m}\frac{r}{a}\right),\;\;\;\;\;r\leq a\\ c_{m}^{\left(2\right)}\;K_{0}\left(w_{m}\frac{r}{a}\right),\;\;\;\;\;r>a \end{array}$$

where a is the radius of the core of the waveguide; $u_{m}$ and $w_{m}$ are the transverse wavenumbers, explicitly defined below; and $J_{0}$ and $K_{0}$ are the Bessel function of the first kind and the modified Bessel function of the second kind, respectively. The super indices of $c_{m}$ indicate the region of the core and the cladding, respectively. In this way, as the light propagates in the MMF section, the total field at the propagation distance z can be calculated as the coherent superposition of all the modes as:

(4) $$E\left(r,z\right)=\underset{m}{\sum }c_{m}F_{m}\left(r\right)\exp\left(-i\beta_{m}z\right)$$

where $\beta_{m}$ is the propagation constant of the m-th mode excited in the MMF. 

Rigorously, the field excitation coefficients, $c_{m}$, are calculated by the overlap integral between $F_{m}\left(r\right)$ and the excitation field $E^{\left(in\right)}\left(r,0\right)$. Alternatively, they can also be estimated through the power coupling coefficient, $\eta_{m}$. For the case of radially symmetric modes, when the input field covers a small region of the core of the multimode waveguide, such that the amount of field in the cladding can be neglected, $c_{m}$ can be related directly to $\eta_{m}$ as modes [11]:

(5) $$c_{m}=\{\begin{array}{c} c_{m}^{\left(1\right)}=\sqrt{\eta_{m}}\\ c_{m}^{\left(2\right)}=\left[\;J_{0}\left(u_{m}\right)/K_{0}\left(u_{m}\right)\right]c_{m}^{\left(1\right)} \end{array}$$

where $\eta_{m}$, in turn, can be estimated as follows:

(6) $$\eta_{m}=\frac{2{\left(\frac{w}{a}\right)}^{2}exp\left[-\frac{1}{2}\left(\frac{w^{2}}{a}\right){\left(u_{m}\right)}^{2}\right]}{J_{0}^{2}\left(u_{m}\right)+J_{1}^{2}\left(u_{m}\right)+\left[\frac{K_{1}^{2}\left(w_{m}\right)}{K_{0}^{2}\left(w_{m}\right)}-1\right]J_{0}^{2}\left(u_{m}\right)}$$

with

$$u_{m}=\left(2m-\frac{1}{2}\right)\frac{\pi }{2}$$

(7) $$w_{m}=\sqrt{V^{2}-u_{m}^{2}}$$

$$V=\left(\frac{2\pi }{\lambda }\right)a\sqrt{n_{r}^{2}-n_{c}^{2}}$$

where $n_{r}$ and $n_{c}$ are the RI of the core and the cladding of the MMF, respectively. Moreover, for a step-index MMF, the propagation constant $\beta_{m}$ of the *m*-th mode can be estimated as [9]:

(8) $$\beta_{m}\approx k_{0}n_{r}-{\left(2m-\frac{1}{2}\right)}^{2}\frac{\pi^{2}}{8k_{0}n_{c}a^{2}}$$

where $k_{0}=2\pi /\lambda_{0}$ is the magnitude of the free-space weave vector and $\beta_{m}$ can also be expressed in terms of the effective RI of the *m*-th mode as $\beta_{m}=k_{0}n_{eff,m}$. 

For the case of waveguides with other geometries, e.g., planar/rectangular, the steps in the approach outlined here would be similar in terms of (i) calculating the energy coupling to each mode supported by the multimode waveguide, (ii) estimating the corresponding propagation constant of each mode, and (iii) constructing the total field as the coherent superposition of all the modes, each traveling with its propagation constant. However, the specific field distribution of the modes supported by the multimode waveguide of interest must be considered for proper calculations [9,10].

In addition, the approach outlined in this work, which was done by following Refs. [11,15], is based on a formulation given in terms of the normalized frequency and the normalized transverse wavenumbers (Equation (7)). These quantities, by definition, are implicit functions of the RI of the cladding of the MMF. Recently, an effort has been made to provide more refined analytical expressions for the effective propagation constant of each mode in the MMF, e.g., using the so-called “far from cutoff” approximation [16,17]. These new expressions are equivalent to the ones described here, but the RI of the cladding of the MMF appears explicitly in the estimations of $\beta_{m}$.

### 2.2. Self-Imaging Properties

Intuitively, in any case where MMI phenomena are involved, one can anticipate that the length of the MMF plays a major role in the outcome. For the case of restricted, symmetric interference, single images of the input field are produced at effective distances $L=p\left(3L_{\pi }/4\right)$, where p=1,2,3,… and $L_{\pi }$ is the so-called beating length between the two lowest-order modes [9]. 

This beating length can be estimated as $L_{\pi }\approx 4n_{eff}W_{eff}^{2}/\left(3\lambda_{0}\right)$, with $n_{eff}$ and $W_{eff}$ being the effective RI and effective optical diameter, respectively, of the lowest-order mode. In a zeroth-order approximation, $n_{eff}$ and $W_{eff}$ can be taken as the RI of the MMF core, i.e., $n_{eff}\approx n_{r,MMF}$, and the geometrical diameter of the core of the MMF, i.e., $W_{eff}\approx W_{MMF}$, respectively. 

**Figure 3:**
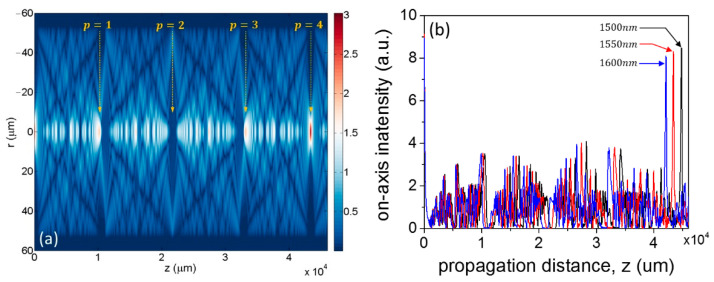
(**a**) Field intensity of the MMI pattern in an SMF-MMF arrangement. The structure was simulated at a free-space wavelength of 1550 nm for SMF/MMF with core diameters of a = 8.6/105 µm and numerical apertures of NA = 0.12/0.22, respectively. The distance z indicates the propagation distance in the MMF, i.e., z = 0 represents the interface between the SMF and the MMF. (**b**) On-axis (r = 0) intensity for the same structure at different free-space wavelengths (1500, 1550, and 1600 nm) showing a sharp image of the input field at p=4.

Just as an example, following the formalism outlined in Section 2.1, in Figure 3a, we show the calculation of the so-called interference carpet, at $\lambda_{0}=1550\;\mathrm{nm}$, for an SMF-MMF structure consisting of an SMF with a core diameter d=8.2 µm and numerical aperture NA=0.14, and an MMF with d=105 µm, NA=0.22, and L=46 mm. The core sizes and numerical apertures are typical values of commercially available fibers. Figure 3a shows the intensity of the total field, $I\left(r,z\right)={\left|E\left(r,z\right)\right|}^{2}$, calculated from the superposition of all the modes in Equation (4). Once again, because the SMF and the MMF are coaxial, and the input field imposes a radial symmetry, the modes excited in the MMF are a subset corresponding to the family of ${\mathrm{LP}}_{0m}$ modes [11,15]. The relative contribution of each mode is determined by the energetic content resulting from the overlap with the input field (Equation (6)). As they propagate with their propagation constant, all the excited modes interfere, creating the interference pattern shown in Figure 3a, typical of MMI devices operated under self-imaging conditions. Integer values of the self-image index p are indicated, which correspond to integer multiples of $L_{\pi }$. The fourth image (p=4) replicates the configuration of the input field, while the first and third images produce a broader version of it, and the second image results in a situation of destructive interference on-axis [9,10].

Figure 3b shows the on-axis intensity, i.e., horizontal cut in Figure 3a at r=0, for the three wavelengths of 1500, 1550, and 1600 nm, respectively. Figure 3 provides the first clear hint of the typical filter-like spectral response of an MMI device: Only one wavelength can produce a replica of the input field on-axis for a fixed propagation distance. In other words, if a second SMF is placed at the output, only one wavelength will result in maximum transmission, as discussed next. 

### 2.3. Spectral Response

**Figure 4:**
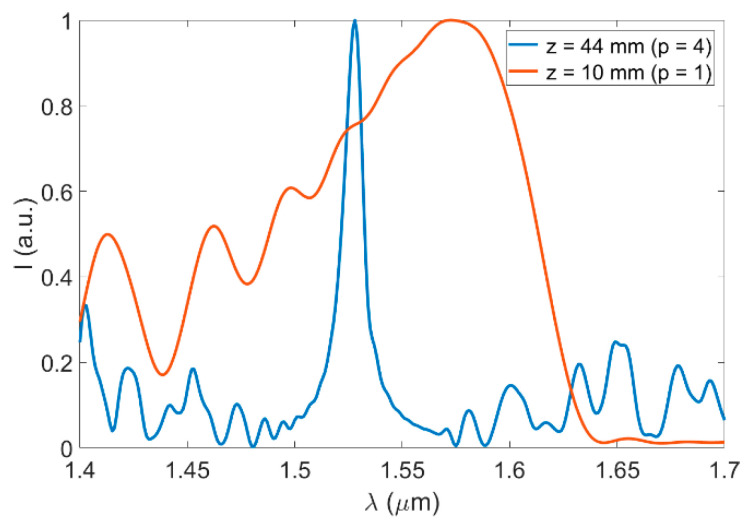
Normalized spectral response of an SMF-MMF with SMF/MMF with core diameters of a = 8.6/105 µm and numerical apertures NA = 0.12/0.22, respectively. The two curves show the output intensity at different propagation distances z, as indicated, which correspond to the first image (p=1) and fourth image (p=4) of the MMI structure.

In Figure 3a, there are replicas sharper than others [15]. In most applications, including imaging, sensing, and laser tuning, these integer replicas are targeted to take advantage of a better resolution in space or, equivalently, a narrower spectral response. For instance, in spectrally operated devices, e.g., for laser tuning applications, it has been shown that the fourth image (p=4), and multiples of it, provide significantly narrower spectral filtering than p=1 [13]. This is shown in Figure 4, where we illustrate the transmitted spectrum through an SMF-MMF structure, at two different propagation distances, which correspond to the first and fourth image, respectively. Each curve was normalized to its maximum for better visualization, but as shown in Figure 3b, the amplitude of the first image is significantly lower than that of p=4.

## 3. MMI-Based Fiber-Optic Sensors

The filter-like spectral response of fiber-optic MMI devices under conditions of symmetric interference, namely when the MMF is excited at its center by a radially symmetric input field, is governed by the following expression [9]:

(9) $$\lambda_{peak}=p\frac{n_{eff}W_{eff}^{2}}{L}$$

This expression allows calculating the peak wavelength $\lambda_{peak}$ that will replicate the *p*-th image of the input field in an MMI device of length L, effective RI $n_{eff}$, and effective optical diameter $W_{eff}$, which can be estimated by correcting the physical diameter, W, with the penetration depth of the evanescent tails into the cladding as [9,10]:

(10) Weff=W+12(λ0π)(nr2−nc2)−12[(ncnr)2+1]

where again, $n_{r}$ and $n_{c}$ are the RIs of the core and the cladding, respectively, of the MMF, $\lambda_{0}$ is the free-space wavelength, and W is the geometrical diameter of the MMF. Strictly, $n_{r}$, $n_{c}$, and $W_{eff}$ are implicit functions of $\lambda_{0}$.

For most practical situations, the second term in Equation (10) represents a correction smaller than one wavelength. This means that the penetration of the evanescent field into the cladding is practically negligible. Therefore, for the case where MMF with a core much larger than the wavelength is used, the approximation $W_{eff}\approx W$ holds as a good estimate. At the same time, this imposes some limitations to manipulate $W_{eff}$ optically in a significant way. In other words, $W_{eff}$ can be altered optically only for smaller values of W, that is, for thin MMFs. Indeed, if the diameter of the MMF is reduced, two effects contribute to make the evanescent penetration deeper overall: If W is reduced, the second term in Equation (10) becomes significant and, at the same time, the confinement factor decreases due to the smaller core, thus giving rise to longer evanescent tails [18,19,20]. Based on this, a typical approach to enhance the sensitivity to the surroundings relies on either tapering or etching down the MMF diameter to produce longer evanescent tails, as will become evident later.

### 3.1. Conventional Sensing (RI, Temperature, Displacement, and Strain)

Equation (9) provides explicit hints for the physical variables that can be sensed directly, such as RI, displacement, and strain. For instance, if one changes the RI of the core or the cladding of the MMF, without altering the geometrical parameters, then a change in $\lambda_{peak}$ will be determined by the change induced to $n_{eff}$. Ultimately, $n_{eff}$ is determined by both $n_{r}$ and $n_{c}$, and both can be manipulated in the experiments, for instance, by replacing them with the liquid sample whose RI is of interest.

In this regard, a typical approach that has become popular consists of using a so-called no-core fiber (NCF) as the MMF in the MMI device. This will become evident in the upcoming sections. Simply put, an NCF is an optical fiber without a core, only a solid silica rod, whose cladding is the medium surrounding it. With this simple approach, variations in the RI of the medium in which the NCF is immersed can be monitored. This approach has been used in many different scenarios, for instance, to measure the RI of different liquids and the composition of binary liquid mixtures from the RI reported, and to measure the liquid level as the NCF is progressively covered [14].

Moreover, by taking advantage of the thermal expansion effects being small in silica, one can exploit the thermo-optic ones to measure the temperature indirectly through variations in the RI of the MMI device. Following up on the same examples where an NCF is used, the only requirement to measure the temperature would be to immerse the MMI device in a calibrated liquid with a sizeable TOC, e.g., Cargille RI oils. NCF-based approaches are so popular for gaining sensitivity to the fiber surroundings that the application of NCFs for different purposes has been the subject of a recent review [21].

From Equation (9), one can see that a change in $\lambda_{peak}$ can also be induced by modifying L, which in turn, can be altered by inducing some strain. In the case of a liquid MMF, this can be achieved by merely displacing the output SMF. Moreover, by knowing the response to strain, these types of sensors can be easily transformed into displacement sensors.

Finally, one can also note that an even more significant change in $\lambda_{peak}$ could also be induced by modifying $W_{eff}$ due to the quadratic dependence. $W_{eff}$ can be altered both geometrically and optically (see Equation (10)). Unfortunately, exploiting this parameter requires overcoming some fundamental limitations, such as the fact that in most situations, $W_{eff}\approx W$, and the fact that in silica, both thermal expansion and elasto-optic effects are, in general, small. These issues will be addressed in more detail in upcoming sections.

All the scenarios mentioned above for sensing conventional physical variables have already been approached in detail in Ref. [14]. Once again, the present review focuses on MMI fiber sensors for nonconventional physical variables. We also refer the readers to a recent review where the fabrication of a variety of MMI architectures has been summarized [22]. In such a review, the main fabrication processes reported in the literature are explained in detail, and most of them apply to the sensors covered in the present work. Here, we provide details of the fabrication only in cases where customized approaches are involved.

We should also briefly mention some important aspects of the signal processing involved in the operation of MMI devices, which are simple. MMI fiber-optic sensors can be either spectrally operated or intensity-based. In spectrally operated devices, one measures the transmitted/reflected spectrum as a function of the control variable. Then, the typical approach consists of following a reference feature of the spectrum, e.g., maxima or minima, to construct a plot of the wavelength shift versus the control variable; from this plot, the sensitivity can be estimated as the local slope, usually in the linear portions of the curve. In the case of intensity-based devices, one measures the transmitted/reflected intensity, often at a single wavelength, as a function of the parameter of interest. Similarly, the sensitivity is estimated from a plot of the intensity versus the parameter of interest. Regardless of the type of sensor, it is common to find signal processing approaches like denoising, smoothing, or fitting. This depends mainly on the quality of the instruments used and the particular conditions of the measurements. Throughout this review, the readers should keep this kind of basic signal processing in mind unless stated otherwise. The ‘nonconventional’ aspect in our review refers to the physical variables sensed and how the MMI devices are used, not to the signal processing involved, which is standard.

### 3.2. MMI-Based Mechanical Sensing

#### 3.2.1. Vibration

In 2001, Sun et al. [23] demonstrated a so-called optical microphone, another name sometimes given to an optical vibration sensor, operating in transmission. In their implementation, acoustic vibrations are measured by employing an SMF-MMF-SMF structure attached to an aluminum membrane, which provides the means to transfer acoustic vibration to the fiber structure. The acoustic vibration is measured through the transmission losses of the SMF-MMF-SMF structure, and operation in the audio range up to 20 kHz is demonstrated. Moreover, high sensitivity is claimed as human voice can be picked up within 2 m. It should be noted that in the case of vibration sensors, the intensity detected needs to be Fourier-transformed in order to disclose the spectrum of vibrations. This is the case in all sensors covered in this section unless stated otherwise.

A similar structure was presented by Wu et al. [24], a few years later, where the sensitivity was improved by U-bending the MMF. In these conditions of enormous losses, the vibration acting on the fiber structure will change the bending radius, thus modulating the transmitted optical power intensity. This sensor can detect both vibration frequencies and amplitudes over a broad range with good sensitivity, up to 12 kHz.

Guzman-Sepulveda et al. [25] presented an MMI vibration sensor operating in reflection (SMF-MMF structure). The MMF section plays the role of the sensing head, in the form of a cantilever, to perform direct vibration measurements, and its facet was coated with a gold layer to improve the intensity of the reflected optical signal. The sensor was tested by characterizing its bending response, its impulse response, and its response to fixed external frequencies up to 2.5 kHz. A clean measurement is reported where the frequencies of interest overcome the first and second harmonics by 32.4 and 37.2 dB, respectively.

In 2014, another MMI vibration sensor based on an SMF-MMF-SMF structure and operating in transmission was presented [26]. The SMF-MMF-SMF structure is attached to a vibrating cantilever beam, which in turn, is attached to a fixed bracket from one end and has a permanent magnet glued to the other. In this case, the setup was more sophisticated. On the one hand, the vibration source was based on the interaction between the electromagnetic coils and the permanent magnet fixed to the cantilever beam. On the other hand, lock-in detection was used in order to measure cleaner signals. Unfortunately, only operation in the narrow range from 2 to 80 Hz was demonstrated.

Ran et al. [27], in 2015, presented an NCF-based MMI vibration sensor attached to a cantilever slab, and operation in transmission was reported. The sensor is glued to the vibrating end of the cantilever slab, and there is a significant increase in the measurement range. In this case, through intensity demodulation, the vibration sensing structure can detect continuous vibration disturbances ranging from 100 Hz to 29 kHz. The frequency sensing resolution is 1 Hz in real-time monitoring.

In 2017, another MMI vibration sensor based on an SMF-MMF-SMF structure and operating in transmission was presented [28]. In this case, the MMF is long (about 30 cm), and operation is not performed under self-image conditions. Still, modulation of the beating between the different modes is achieved when the MMF vibrates. Measurements in the low-frequency range from 30 to 180 Hz were demonstrated.

The sensitivity of an MMI vibration sensor consisting of an SMF-MMF-SMF structure was enhanced by tapering the MMF [29], and then their implementation was further improved using Helmholtz resonators [30]. After these optimizations and implementations of sensitivity enhancement mechanisms, the new reports focused more on the applications rather than proof-of-concept demonstrations. For instance, in recent reports, MMI vibration sensors were used to detect partial discharges in high-power electrical machinery [31] and monitor the heart rate [32].

One of the main observations in this area is that most of the reports work with configurations in transmission and that an intermediate transducer is typically used to transfer the vibrations to the optical fibers. Only one report exists where the operation is in reflection, and the optical fiber is made to vibrate directly, playing the role of the cantilever beam itself [25]. We also noted that MMI vibration sensors had recently been put to work in practical situations.

#### 3.2.2. Pressure

The first pressure sensors based on MMI phenomena were optomechanical semiconductor structures where MMI splitters/couplers, either with silica waveguides [33] or polymer waveguides [34], were inscribed on a thin membrane. When no pressure is applied to the membrane, the output coupler reports the constructive interference of the two divided paths. On the other hand, when some pressure is applied, the membrane deflects, producing a phase difference between the two paths due to path lengthening (strain) and the photo-elastic effects (change in the effective RI due to the stress applied).

Similar mechanisms govern the operation of the first optical fiber-based MMI sensors reported. However, it was not until ten years later that they were reported [35]. In their sensing scheme, Ruiz-Perez et al. glued an NCF-based MMI sensor to a 6 mm-thick acrylic that played the role of a window of a pressure chamber. When some pressure is applied, the acrylic plate deflects, thus producing a shift in the MMI image, which in turn, produces a spectral shift or, equivalently, a change in intensity. In their experiments, the intensity decreases with increasing pressure, which is consistent mainly with bending losses. The sensitivity reported is 4 mV/psi over the range from 0 to 140 psi. They also report an enhanced sensitivity of 35 mV/psi when a 3 mm-thick acrylic is used; however, the operation range reduces drastically to only 10 to 30 psi. A few years later, Mejía-Aranda et al. reported an improvement over this work by extending the measurement range up to 100 psi [36]. The response is nonlinear, with sensitivities of 3.86 and 1.52 mV/psi, in the range from 0 to 37 psi and from 37 to 100 psi, respectively.

Ruiz-Perez et al., in 2011, reported a new type of pressure sensor: An NCF-based MMI sensor partially covered by a thin membrane of the polymer PDMS [37]. As pressure increases, the membrane is pushed toward the NCF, thus covering a larger portion of the periphery of the NCF. In this case, the principle of operation relies on a change in the effective RI of the cladding of the NCF, by partially covering it by different amounts that depend on the pressure applied. The sensitivity reported is 3 μV/psi in the range from 0 to 60 psi. A few years later, in 2016, the same work was extended to an operating range up to 140 psi, reporting a similar sensitivity of −1 µW/psi [38].

Although the improvement in both the operation range and the sensitivity is evident over time, pressure sensing finds some limitations. For instance, typically, the optical fiber needs to be attached to a deformable membrane, which imposes some restrictions on both the range and the sensitivity that can be achieved: First, due to the natural trade-off between the deformability and the operation range and, second, due to the stress transfer from the membrane to the optical fiber whose photo-elastic coefficient is low. In this regard, a possible solution could be the use of plastic optical fibers that can sustain larger deformations. Table 1 summarizes the main parameters that describe the performance of MMI pressure sensors.

### 3.3. MMI-Based Electromagnetic Sensing

#### 3.3.1. Magnetic Field

MMI structures have been used for magnetic field (MF) sensing, where usually ferromagnetic fluids play the role of opto-magnetic transducers. The effective optical properties of the fluid, which consists of a suspension of magnetic field-responding nanoparticles, change depending upon the external MF. The typical transducing mechanism involves the orientation of the suspended nanoparticles with the MF, which results in variations in the effective RI of the fluid, similarly to how a liquid crystal responds to an applied voltage.

Most of these sensors consist of an SMF-MMF-SMF architecture, where an NCF is used as the MMF. The structure is surrounded by the ferromagnetic fluid, for instance, by immersing the structure in a sealed capillary tube. Variations exist on the type of MMF, i.e., square or cylindrical NCF; the mode of operation, i.e., transmission/reflection or intensity-based/spectral; and the mechanisms implemented to improve the sensitivity, e.g., etching, tapering, or nanocoating.

Reviewing the state of the art, we observed that the first related works appeared in 2013, when Lin et al. [39] used an MMI sensor formed by a section of square NCF spliced between two SMFs immersed into the magnetic fluid. Their sensor is intensity-based with a negligible spectral shift. Operation over the range from 0 to 50 mT with a maximum sensitivity of −0.1939 dB/mT was demonstrated.

In the same year, Wang et al. [40] reported a similar structure. Compared to the previous work, they improved the sensitivity by wet etching the NCF, and therefore, the sensor could be operated spectrally. They immersed the structure in an oil-based suspension of ferromagnetic nanoparticles with a saturation magnetization of 10 mT by Ferrotec. Although they operated their setup with an external MF up to 45 mT, the measurement shows saturation beyond 32 mT (meaning the magnetic fluid reached its saturation magnetization). In general, a dose–response type of nonlinear response is obtained in the measurements. The maximum sensitivity, calculated in the linear portion of the curve, was −168.6 pm/mT.

In the same year, Chen et al. [41] also reported a similar MMI device consisting of a fusion-spliced section of NCF between two SMFs. The magnetic fluid was a water-based suspension of ferromagnetic nanoparticles with a saturation magnetization of 22 mT by Ferrotec. They did not implement any enhancing mechanism, but they chose a fluid whose RI is around 1.4 in the absence of external MF, which naturally produces long evanescent tails due to the proximity to the RI of the NCF. The nonlinear response is obtained in the measurements too; the linear portion goes from 4 to 10 mT. In this region, they report a spectral sensitivity of 905 pm/mT and intensity-based sensitivity of 0.748 dB/mT.

One year later, Zhang et al. [42] also reported a similar NCF-based MMI structure. Compared to the previous reports, they also used similar water-based magnetic fluids, with an RI close to that of the NCF, but the sensitivity was increased further by U-bending the section of NCF, which produced a deeper evanescent penetration. Their measurements also show a nonlinear response, especially at low MF. When the sensor is bent, they report a sensitivity of 3185.2 pm/mT, ranging from 2 to 10 mT. For reference, they also performed measurements with the sensor straight, reporting a sensitivity of 275.7 pm/mT, in the range from 0 to 5 mT, and 742.9 pm/mT, in the range from 5 to 10 mT.

Finally, in 2015, Ascorbe et al. [43] reported a similar NCF-based MMI device, but they proposed a novel approach: Instead of using magnetic fluids, they deposited a ferromagnetic nanocoating around the NCF consisting of a cobalt-nickel magnetic alloy. They operated their setup from 0 to 330 mT and reported spectral and intensity-based sensitivities of 6 nm/T and 1.45 dB/T, respectively. As can be noted, the sensitivity was much lower than in previous reports; however, this approach removes the need for a ferromagnetic fluid and its associated issues, e.g., slow response and evaporation, and it also facilitates the fabrication as extra steps after coating are not required.

Overall, the following limitations are noted for MF fiber-optic sensors based on MMI phenomena. First, even with the implementation of sensitivity-enhancing mechanisms, practically all works report a small sensitivity (except for the U-bent sensor from Zhang et al. [42], 3.19 nm/mT). The nonlinear response also exhibits negligible amplitude at low MF and saturation at high MF, which leaves only a limited range for linear operation. Finally, due to the slow response of magnetic fluids, all the MF sensors listed here have only been used for measuring static fields. We anticipate that with the advent of better magnetic fluids with larger saturation magnetization and faster response [44,45], most of the current limitations can be overcome without changing the NCF-based MMI structure. It is also interesting to note that other sensitivity-enhancing mechanisms, like tapering, or the combination with other fiber structures, have not been explored. Table 2 summarizes the main parameters that describe the performance of MMI sensors for MF sensing.

#### 3.3.2. Electric Current

Following up on the previous section, having MMI structures sensitive to MFs makes them readily available for sensing electric current. Nevertheless, to the best of our knowledge, there exist only a couple of reports of MMI current sensors. In Ref. [46], Li et al. reported a typical SMF-MMF-SMF structure, with a piece of NCF as the MMF, sealed in a capillary filled with magnetic fluid. The sensor is sensitive to the surrounding MF but, with an initial characterization of the setup to reveal the relation between the MF and the electric current induced, the optical measurement can be converted to retrieve the electric current. They report a sensitivity of 1.08 V/A, in the range from 0 to 10 A. The same group reported a similar structure operated in reflection [47]. In this second report, a more detailed characterization was performed. Specifically, the sensitivity was tuned by misaligning the sensor controllably with respect to the external MF. Due to double pass of the reflection configuration, the sensitivity doubles, reporting a maximum sensitivity of 2.18 V/A, in the same range from 0 to 10 A. In both cases, they performed their measurements in a ratiometric manner by using balanced detectors. Due to the negligible response of the magnetic fluid at low MFs, the sensor exhibits a minimum operation threshold of a few hundred milliamperes in both cases.

#### 3.3.3. Voltage

To the best of our knowledge, only one report exists where an MMI structure has been used for voltage sensing [48]. The sensor reported consists of a typical SMF-MMF-SMF structure that is glued to a piezoelectric crystal. In this way, voltage is inferred indirectly from the strain response of the MMI sensor. The measurement was performed in a ratiometric scheme with a self-reference optical power measurement, and a resolution of about 0.5 V is demonstrated.

This is an open area that has not been explored yet. We anticipate that, by using plastic optical fibers that can sustain large deformations and respond faster to strain, the sensitivity could be enhanced, and the application to sensing dynamic voltage could be possible.

### 3.4. MMI-Based Chemical Sensing

#### 3.4.1. Relative Humidity

Relative humidity (RH) sensors based on the MMI effect consist of two principal parts. The first one is the fabrication of the optical fiber sensing platform, and the second one is the deposition of the moisture-sensitive coating material. Below, we present the most relevant works in this area.

In 2011, Zhao et al. [49] presented an RH sensor based on an SMF-MMF-SMF fiber structure. A thin layer of polyvinyl alcohol (PVA) was used to coat the MMF. For increasing RH sensitivity, a solution of hydrofluoric acid was used to wet-etch the cladding of the MMF, whose RI varies as a humidity level. The sensitive material was PVA due to its appropriate humid sensitivity. The RH measurement sensitivity is 0.18 nm/% RH in the range from 80 to 89% RH.

Later, in 2013, Li et al. [50] also presented an RH with an NCF-based SMF-MMF-SMF structure. This time, a combination of hydroxyethyl cellulose (HEC) and polyvinylidene fluoride (PVDF) works as a moisture-sensitive material forming the hydrogel coating on the NCF by the dip impregnation method. The humidity-induced RI changes outside the NCF, leading to variations in the output optical power. The structure of 2 cm NCF achieved a sensitivity of 0.196 dB/%RH, at 1310 nm, with a better response when the RH was lower than 75%.

In 2013, the same research group of Ref. [49] presented a similar RH sensor with an SMF-MMF-SMF structure [51]. In this work, the operating range improved. It was from 30 to 80% RH. The wavelength sensitivity and intensity sensitivity changed to 0.9 nm/%RH and 0.3 dB/%RH, respectively.

An et al. [52], in 2014, presented a sensitivity enhancement over the RH sensor presented in Ref. [49]. The main difference consists of the implementation of bitapers at both ends of the MMF. The bitaper consists of two waist-enlarged tapers that enhance the coupling efficiency of the SMF-MMF-SMF fiber structure. PVA was selected as the suitable sensitive material due to its appropriate humid sensitivity. The same operation principle applies here; the PVA RI showed a redshift wavelength as its humidity varied. The sensitivity of this sensor was 0.139 nm/%RH of the wavelength at 1506.7 nm (dip A) and 0.223 nm/%RH of the wavelength at 1537.6 nm (dip B). The RH range was from 35% to 85%.

In 2016, Miao et al. [53] proposed a low-temperature-sensitive RH sensor based on the taper square NCF (TSNCF) coated with SiO_2_ nanoparticles. They used the SMF-MMF-SMF structure as in the previous works. The sensor was fabricated by splicing a section of TSNCF between two standard SMFs, and then it was immersed into a suspension of SiO_2_ nanoparticles that deposited on the surface of the TSNCF. The RI of the SiO_2_ nanoparticles changed with the variations in the environmental humidity levels. These variations led to a two-dip (dip A around 1410 nm and dip B around 1610 nm) spectral redshift. The range reported was from 43.6 to 98.6%RH, but it was also mentioned that the SMF-MMF-SMF structure of 2.7 cm TSNCF achieved its best sensitivity from 83 to 96.6%RH. It was 456.21 and 584.2 pm/%RH for the two dips.

In the same year, Xu et al. [54] reported an RH sensor based on an SMF-MMF-SMF structure with an NCF as the MMF. This time, an agarose gel film (AGF) coated the NCF by dip-coating technology with different concentrations and different coating numbers, varying the AGF effective RI. The RH could be measured indirectly through the RI changes of the coating film. The wavelength and intensity sensitivity obtained was −149 pm/%RH and −0.075 dB/%RH, respectively, ranging from 30% to 75% RH.

Finally, Wang et al. [55], in 2017, developed a single-mode-side polished multimode-single-mode (SSPMS)-structure RH sensor. The main difference with the SMF-NCF-SMF structure was that the whole cladding and part of the core in the MMF section were removed by polishing to make the remaining fiber structure a D-shaped multimode waveguide, which allows the core section to be directly exposed to the external environment. The multimode part of the structure was coated with three layers of gelatin as the moisture-sensitive film by the dip-coated method. Gelatin consisted of a mixture of peptides and proteins whose optical properties depend on the amount of water trapped in it. The operating principle relies on the transmitted power loss referred to as the multimode D-shape structure and its variation with the RH value of the external environment. The highest sensitivity was 0.14 dB/%RH and a fast response time of 1000 ms for 40–90%RH for a humidity sensing range.

All the RH sensors proposed to this date are based on an SMF-MMF-SMF structure, and most of them use an NCF as the MMF. The SMF-MMF-SMF structure was utilized due to multiples merits such as the ease of fabrication, high sensitivity, and low cost compared with other optical fiber structures. Several moisture-sensitive materials have been applied as coated films to enhance the RH sensitivity sensor. These materials have been PVA, agarose gel, PVDF, SiO_2_ nanoparticles, and gelatin. All the works reviewed measured the RH, in an indirect form: When the surrounding humidity changes, the coating materials exhibit a variation in RI, and hence, the light transmission through the structure is altered in a detectable way, allowing for sensing humidity. All the RH sensors reviewed operate around 1550 nm, offering acceptable real-time RH monitoring and a low-temperature dependence. The main characteristics of the seven RH sensors are summarized in Table 3.

#### 3.4.2. Gas Sensing

Gas sensors based on MMI effects also consist of two principal parts: The fabrication of the optical fiber sensing platform, and the deposition of the gas concentration-sensitive coating material. To the best of our knowledge, only a couple of reports exist in the literature where MMI gas sensors are demonstrated. Below, we present their most relevant aspects.

Li et al. [56], in 2016, presented a reflection-type optical fiber methane sensor using an NCF-based structure. A cryptophane A/polysiloxane composite was performed as a sensitive film on the NCF, and it was deposited by the dip-coating method. An SMF was fusion-spliced to one end of the NCF, and a nickel (Ni) reflecting film was electro-plated at the second end of the NCF. The light that travels from the SMF to the NCF excites multiple modes propagating within the NCF to form interference due to the mode field mismatch. The light reflects at the Ni film and couples back to the SMF. The waveguide parameters vary with the RI changes of the methane-sensitive film. Therefore, the interaction between the interference from the excited modes and the methane-sensitive film will lead to the fringe movement of the reflecting interference spectra. The operation ranges were from 0 to 15% and 1.5 to 3.5% gas concentration. In those ranges, the sensitivity reported was 0.85 and 0.24 nm/%, respectively.

Last year, Feng et al. [57] demonstrated a hydrogen sulfide (H_2_S) gas sensor based on the SMF-MMF-SMF fiber structure. Two thin-core fibers (TCFs) were the SMFs, and an NCF was the MMF. A TiO_2_/ZnO composite performed as a sensitive film, and it was deposited on the NCF by the dip-coating method. As TiO_2_ has a strong adsorption capacity for H_2_S and ZnO has a broad application prospect in pollutant treatment, it enhanced the sensor sensitivity by reuniting both. The TiO_2_/ZnO adsorbs the gas causing the NCF to change its effective RI. This change leads to a central wavelength redshift. The operation ranges were 0–50 ppm, and the sensor sensitivity was 21.26 pm/ppm.

The gas sensors reported make use of an SMF-MMF-SMF structure using an NCF as the MMF. Similar to RH sensors, the SMF-MMF-SMF fiber structure was utilized due to some advantages such as compact structure, easy fabrication, and high sensitivity. Two concentration-sensitive composites have been applied as coated films to enhance the gas sensitivity sensor, cryptophane A/polysiloxane, and TiO_2_/ZnO. Fundamentally, however, it is known that MMI structures are sensitive enough for gas sensing in general [58], and other materials have been used in MMI-integrated devices, e.g., bromocresol purple for ammonia detection [59]. It is also worth noting that gas sensing is typically performed indirectly: When the surrounding gas concentration varies, the effective RI of the sensitive coating changes accordingly. Hence, the light transmission through the SMF-MMF-SMF fiber structure suffers a spectral shift, allowing for gas sensing. All the gas sensors reviewed operate around 1550 nm.

### 3.5. MMI-Based Wavelength Monitoring

To the best of our knowledge, only one report exists where MMI fiber devices have been used for wavelength monitoring by the group of Prof. Farrel [60]. The work is based on edge filters, which are constructed based on SMF-MMF-SMF structures operated at p=1 [61]. In their approach, a single MMI edge filter arises by considering half of a broad spectral response at specific imaging conditions (see Figure 4). Then, by using two of those filters in a configuration such that the long-wavelength edge of one filter overlaps with the short-wavelength edge of the second one, the input light wavelength can be discriminated in a ratiometric-type measurement. The resolution reported was smaller than 10 pm, with a discrimination of around 20 dB, in the wavelength range from 1530 to 1560 nm.

### 3.6. New Trends in MMI-Based Sensing

Recently, MMI-based fiber-optic devices have been extended to new applications. Below, we briefly review these new trends that include MMI-based sensing schemes based on polymer fibers, for wavelength-locking applications, for retrieving the TOC of liquid samples, and for measuring the dynamics of complex fluids.

#### 3.6.1. MMI Sensing Using Polymer Fibers

As mentioned before, in silica, both thermal expansion and elasto-optic effects are small. In polymers, in general, this is not the case. In fact, because of this, in the last years, plastic optical fibers (POFs) have been used to fabricate MMI devices, especially for sensing strain and temperature. In general, the POF-based MMI sensors reported in the literature consist of the typical SMF-MMF-SMF structure. In terms of the fabrication, we should point out that in these structures, the silica SMFs are not spliced to the POFs but glued with optical adhesives. 

Huang et al. [62] reported an MMI strain sensor using a polymethylmethacrylate (PMMA) fiber as the MMF. The sensor was adequate for an extended strain range, from 0 to 20,000 με, but unfortunately, it had a low sensitivity of −1.73 pm/με and a relatively large temperature cross-sensitivity of 33 με/°C.

After this first report, the following ones came out from the group of Prof. Nakamura, using perfluorinated (PFGI) and partially chlorinated (PCGI) POFs. In general, these fibers have a graded RI profile (GI), and the sensor’s sensitivity depends strongly on the length of the fiber.

Using a 1 m-long PFGI-POF with a core diameter of 62.5 µm, Numata et al. [63] reported an MMI structure where they characterized the strain and temperature response. For strain, the sensitivity reported was −112 pm/με, in the operation range from 100 to 500 με. This sensitivity reported for strain was approximately 12.9 and 7.7 times larger than in silica and PMMA, respectively. For temperature, on the other hand, the sensitivity reported was −49.8 nm/°C, in the very narrow range from 26 to 27 °C. This sensitivity reported for temperature was over 1800 and 100 times larger than in silica and PMMA, respectively.

In [64], Numata et al. also performed a similar strain and temperature characterization using a 0.7 m-long PCGI-POF. The strain sensitivity reported was −4.47 pm/με, ranging from 0 to 1000 με. The temperature sensitivity reported was −6.76 nm/°C, ranging from 27 to 28 °C. The strain and temperature sensitivity reported was 0.29 times and over 350 times the sensitivity for silica.

The same group extended their work on PFGI-POFs. First, they performed a more detailed temperature characterization of a 1 m-long PFGI-POF with a core diameter of 62.5 µm, where they report a sensitivity of +7.7 nm/°C at room temperature and a much larger sensitivity at higher temperatures; for instance, +202 nm/°C at around 70 °C [65]. Using a 0.7 m-long PFGI-POF with a core diameter of 62.5 µm, they also demonstrated that operating in reflection provides a similar sensitivity than in transmission [66]. For reflection, they reported a strain sensitivity of −122.2 pm/με, in the range from 50 to 300 με, and a temperature sensitivity of +10.1 nm/°C, in the range from 36 to 37.5 °C. Moreover, they demonstrated a temperature sensitivity enhancement of around three times (from +0.75 to +2.17 nm/°C, in the range from 24 to 30 °C) by implementing a thermal treatment that consisted of annealing the PFGI-POF at 90 °C [67]. Finally, a more detailed strain characterization was also performed using a 0.93 m-long PFGI-POF with a core diameter of 62.5 µm and subjecting different POF sections to strain [68]. The lengths of the strained section were 0.1, 0.2, 0.4, 0.6, 0.8, and 0.93 m; regardless of the length, the same amount of displacement was applied, up to 500 μm. They found that sensitivity was proportional to the strained length with a coefficient of −51.7 pm/με/m; the most considerable sensitivity reported was −48 pm/με, in the range from 0 to 540 με.

Last year, 2020, Leal-Junior et al. reported a fiber-optic intrinsic electromagnetic field sensor based on modal interferometry [69]. The sensor uses a short section of POF sandwiched between two SMFs. They fused the portion of the POF, which gives rise to an oscillatory carbonized path in the optical fiber core. Such carbonization of the core results in a hollow-core POF, and the carbonized section has a higher electrical conductivity than the native fiber. The interaction between an external electromagnetic field and the carbonized and noncarbonized regions leads to variations in the optical fiber parameters. This, in turn, leads to different magneto-optic effects such as Kerr effects that result in changes in the RI, polarization, and shape of magnetostrictive materials. In this configuration, variations in the fused-POF refractive index and shape due to electromagnetic fields applied on the sensing region led to a wavelength shift in the transmitted optical spectrum. The sensitivity obtained was 113.5 pm/mT on a 5 cm fused POF, ranging the electromagnetic field from 0 to 240 mT. The sensitivity increased by increasing the fused POF length.

Overall, sensing with POF-based MMI devices is still an emerging field with several windows of opportunity. For instance, we noted that when using POFs in MMI devices, long pieces of fiber (on the order of 1 m) need to be used to account for appreciable effects. Moreover, the operation range reported is narrow for both strain and temperature. We anticipate that, by using light-guiding polymers that can sustain extreme deformations (see, for instance, Refs. [70,71,72,73]), this field could see the advent of MMI plastic sensors with better performance. Table 4 summarizes the relevant parameters of MMI sensors based on POFs.

#### 3.6.2. Athermal MMI Devices for Measuring the TOC of Liquids

In most practical situations involving silica optical fibers, thermo-optic effects amply dominated over thermal expansion effects. Consequently, the intrinsic thermal response of a simple SMF-MMF-SMF structure cannot be canceled unless additional measures are taken. Nevertheless, this is not the case for a more complex MMI device consisting of a cascade of MMF sections where the thermal effects compensate each other [74,75,76].

The spectral response of an MMI device, Equation (9), can be generalized to the case of an arbitrary cascade with N multimode sections as [76]:

(11) $$\lambda_{peak}=p\overset{N}{\underset{i=1}{\sum }}\left(\frac{n_{eff,i}W_{eff,i}^{2}}{L}\right)\left(\frac{L_{i}}{L}\right)$$

with $\frac{1}{L}\sum_{i=1}^{N}L_{i}=1$. $W_{eff,i}$ is the effective optical diameter of each multimode section, as defined above, in Equation (10). The thermal sensitivity $\frac{\partial \lambda_{peak}}{\partial T}$ of the whole structure is:

(12) $$\frac{\partial \lambda_{peak}}{\partial T}=p\frac{\partial }{\partial T}\left[\left(\frac{n_{eff,i}W_{eff,i}^{2}}{L}\right)\left(\frac{L_{i}}{L}\right)\right]$$

where the condition for temperature independence for the whole MMI cascade can be obtained by setting $\frac{\partial \lambda_{peak}}{\partial T}=0$ [77]:

(13) $$\overset{N}{\underset{i=1}{\sum }}\left\{\left(W_{eff,i}^{2}L_{i}\right)\frac{\partial n_{eff,i}}{\partial T}+2\left(n_{eff,i}W_{eff,i}L_{i}\right)\frac{\partial W_{eff,i}}{\partial T}+\left(n_{eff,i}W_{eff,i}^{2}\right)\left[\frac{\partial L_{i}}{\partial T}-2\left(\frac{L_{i}}{L}\right)\frac{\partial L}{\partial T}\right]\right\}=0$$

Equation (13) expresses a complex inter-relationship between the geometrical and optical parameters of the different multimode sections, as well as their temperature response, for the entire composite to be thermally insensitive. It can be noted that Equation (13) is independent of the value of p, which means that the thermal compensation can be achieved, in a material-based manner, irrespective of whether one operates under self-imaging conditions.

The use of these arguments for athermalization opened the venue to fabricating MMI devices where the spectral response is locked down [76]. This, in turn, led to the development, for instance, of fiber lasers that are practically temperature-insensitive, with a thermal response smaller than 1.0 pm/°C in the temperature range from 25 °C to 100 °C [78].

More recently, similar athermalization procedures were implemented to cancel out the inherent thermal response of an MMI cascade to measure the TOC of liquid samples [77]. In their approach, Ruiz-Perez et al. easily achieved a thermo-optically compensated MMI device by merely coating a portion of an NCF with the polymer PDMS in an NCF-based SMF-MMF-SMF configuration. Full thermo-optic compensation was realized by covering an appropriate length of the NCF, and they provided analytical expressions for the design of two-element athermal MMI cascades, i.e., derived from Equation (13) for N=2. From a practical perspective, this approach constitutes a simpler methodology for measuring the TOC of liquid samples because, thanks to the cancellation of the inherent thermal cross-sensitivity, only one calibration measurement is required. 

In both cases (wavelength-locked laser and TOC sensor), the experimental demonstration of the thermal compensation was performed, as explained in Section 3, by monitoring the spectral shift as a function of the temperature, and the sensitivity was estimated from the slope of such a curve. 

#### 3.6.3. Dynamic Light Scattering Assisted by Fractional MMI Phenomena

Finally, MMI effects have also been used to assist passive optical sensing schemes. Contrary to the sensing applications mentioned in previous sections, in this case, MMI phenomena help to carry information, but the MMF section, where MMI takes place, does not act as a transducer. In particular, MMI effects were used to improve a so-called low-coherence dynamic light scattering (LC-DLS) technique, which is implemented on an all-fiber platform to measure dynamic scattering, from where the dynamics of complex fluids is retrieved [79].

By assisting a single-mode, fiber-based LC-DLS setup with MMI effects, the ensemble dynamics of complex fluids can be measured over effectively larger regions while preserving a signal quality comparable to that of a purely single-mode scheme. Simply put, by assisting the single-mode DLS technique with MMI effects, it is possible to collect light efficiently and interact with the medium over larger areas thanks to the use of an MMF probe, while keeping a high signal quality due to the spatial filtering of the SMF. From a practical standpoint, such a configuration can prevent nonergodic manifestations in a DLS measurement, while the signal quality is preserved.

To the best of our knowledge, Ref. [79] is the only work where fractional fiber-optic MMI devices have been reported [5]. The fractional replicas were generated in the range p≤1. In addition, the results presented constitute the first experimental demonstration of the filtering properties of an MMI device in the direct domain, i.e., length, instead of the spectral domain, i.e., wavelength [12,13]. Moreover, in this work, the MMI device was operated in the visible range of the spectrum as opposed to the widely explored infrared telecom range.

In this case, the operation consists of a standard intensity-based configuration, but the signal processing is more convolved. The intensity detected is random in nature and the core of the information retrieval relies on the power spectrum of this fluctuating signal. From the power spectrum, the dynamic information of the complex fluid can be retrieved to extract, for instance, the hydrodynamic size of the diffusing particles or even the viscoelastic moduli of the suspending medium [80]. A detailed description can be found in Ref. [81].

## 4. Conclusions

In the present work, we reviewed optical fiber-based MMI devices for sensing applications, covering around fifteen years of work in the field. We focused our attention on MMI fiber sensors for nonconventional physical variables, including mechanical, electromagnetic, chemical, and optical. We also reviewed some new sensing schemes, for instance, those based on polymer fibers, for retrieving the thermo-optic coefficient of liquid samples; and for measuring the dynamics of complex fluids. For each section, we summarized the work in the field in a condensed yet informative manner; we provided some explanations about the fundamental aspects of the principle of operation; and we also gave some insights on how the field could move forward, as well as some unexplored possibilities. 

Besides the outlook provided in each section, we identified some areas of opportunity for future work in the field. For instance, MMI sensors have been applied to biomedical situations only in a few instances. Besides the above-mentioned cardiac monitor [32], MMI sensors have been used to monitor breathing based on bending losses [82], and to carry out immunoassays based on changes in the effective RI [83]. In addition, although MMFs have been used in conjunction with plasmonic structures since some time ago [84,85,86], we noticed that the typical configuration involves a primitive measurement of the transmitted intensity through the fiber link. However, the devices are not operated under the MMI conditions covered in this review, like self-imaging. It is worth reminding that operating MMI devices in these conditions makes them optimal in practical applications. 

In closing, as evident from this review, optical sensing based on MMI phenomena is a field in significant growth. It applies to many practical situations, mainly due to the simplicity of the structures used, the ease of fabrication, the considerable sensitivity inherent to MMI, and the universal presence of MMI phenomena across the electromagnetic spectrum. It should be encouraging for scientists and researchers to see that there exist several important areas that remain yet to be explored.

## Figures and Tables

**Table 1:** Summary of the relevant parameters of MMI pressure sensors.

Ref.	Year	Measurement Range	Sensitivity
[35]	2010	0–140 psi (0–965.27 kPa)	4 mV/psi
[37]	2011	0–60 psi (0–413.68 kPa)	3 μV/psi
[36]	2013	0–7 bar (700 kPa, 101 psi)	56 mV/bar (3.86 mV/psi) (0–2.5 bar; 0–37 psi) 22 mV/bar (1.52 mV/psi) (2.5–7 bar; 37–100 psi)
[38]	2016	0–960 kPa (0–140 psi)	−0.145 × 10^−3^ mW/kPa

**Table 2:** Summary of the relevant parameters of MMI magnetic field sensors.

Ref.	Year	Range	Sensitivity
[39]	2013	0–500 Oe (0–50 mT)	−0.01939 dB/Oe (−0.1939 dB/mT)
[40]	2013	0–450 Oe (0–45 mT)	−16.86 pm/Oe (−168.6 pm/mT)
[41]	2013	0–220 Oe (0–22 mT)	90.5 pm/Oe (905 pm/mT) 0.748 dB/mT
[42]	2014	0–100 Oe (0–10 mT)	275.7 pm/mT (straight; 0–5 mT) 742.9 pm/mT (straight; 5–10 mT) 3185.2 pm/mT (U-bend; 2–10 mT)
[43]	2015	0–330 mT	1.45 dB/T 6 nm/T

**Table 3:** Summary of the relevant parameters of MMI relative humidity sensors.

Ref.	Year	Range	Sensitivity
[49]	2011	80–89%RH	0.18 nm/%RH
[50]	2013	40–90%RH	0.015768 dB/%RH (0.5 cm NCF) 0.1359 dB/%RH (1.0 cm NCF) 0.196 dB/%RH (2.0 cm NCF)
[51]	2013	30–80%RH	0.09 nm/%RH 0.3 dB/%RH
[52]	2014	35–85%RH	0.139 nm/%RH (dip A) 0.223 nm/%RH (dip B)
[53]	2016	43.6–98.6%RH 83–96.6%RH	456.21 pm/%RH (dip A) 584.2 pm/%RH (dip B)
[54]	2017	30–75%RH	−149 pm/%RH −0.075 dB/%RH
[55]	2017	40–90%RH	0.14 dB/%RH

**Table 4:** Summary of the relevant parameters of MMI sensors based on plastic optical fibers.

Ref	Variable	Range	Sensitivity
[62]	Strain	0–20,000 με	−1.73 pm/με
[63]	Strain Temperature	100–500 με 26–27 °C	−112 pm/με −49.8 nm/°C
[64]	Strain Temperature	0–1000 με 27–28 °C	−4.47 pm/με −6.76 nm/°C
[65]	Temperature	10–70 °C	+7.7 nm/°C (at RT) +202 nm/°C (around 70 °C)
[66]	Strain Temperature	50–300 με 36–37.5 °C	−121.8 pm/με (transmission) −122.2 pm/με (reflection) +9.63 nm/°C (transmission) +10.1 nm/°C (reflection)
[67]	Temperature	24–30 °C	+2.17 nm/°C (annealed) +0.75 nm/°C (native)
[68]	Strain	0–540 με	−48 pm/με
[69]	Magnetic field	0–240 mT	113.5 pm/mT

## Data Availability

Not application.
